# Transient Response and Firing Behaviors of Memristive Neuron Circuit

**DOI:** 10.3389/fnins.2022.922086

**Published:** 2022-06-22

**Authors:** Xiaoyan Fang, Yao Tan, Fengqing Zhang, Shukai Duan, Lidan Wang

**Affiliations:** ^1^College of Artificial Intelligence, Southwest University, Chongqing, China; ^2^Department of Big Data and Machine Learning, Chongqing University of Technology, Chongqing, China

**Keywords:** memristor, neuron, RC circuit, MC circuit, firing behaviors

## Abstract

The signal transmission mechanism of the Resistor-Capacitor (RC) circuit is similar to the intracellular and extracellular signal propagating mechanism of the neuron. Thus, the RC circuit can be utilized as the circuit model of the neuron cell membrane. However, resistors are electronic components with the fixed-resistance and have no memory properties. A memristor is a promising neuro-morphological electronic device with nonvolatile, switching, and nonlinear characteristics. First of all, we consider replacing the resistor in the RC neuron circuit with a memristor, which is named the Memristor-Capacitor (MC) circuit, then the MC neuron model is constructed. We compare the charging and discharging processes between the RC and MC neuron circuits. Secondly, two models are compared under the different external stimuli. Finally, the synchronous and asynchronous activities of the RC and MC neuron circuits are performed. Extensive experimental results suggest that the charging and discharging speed of the MC neuron circuit is faster than that of the RC neuron circuit. Given sufficient time and proper external stimuli, the RC and MC neuron circuits can produce the action potentials. The synchronous and asynchronous phenomena in the two neuron circuits reproduce nonlinear dynamic behaviors of the biological neurons.

## 1. Introduction

All organisms in a dynamic environment can interact with each other mainly because the brain can efficiently process and propagate the information flow. The brain is composed of neurons, and the information transmission between neurons is characterized by spiking behaviors (Kumar et al., 2013). The spiking patterns generated by biological neurons help us better understand how cortical neurons work. With the development of artificial general intelligence (Li et al., 2021; Yang et al., 2022a,b) and its hardware (Yang et al., 2020, 2021), the simulation of the structure and function of neurons in the brain has attracted great attention in various fields. Especially the study on the fundamental circuits of neurons, a lot of studies on the RC circuit have focused on the filter (Caves et al., 1977; Chen et al., 2021), the oscillator (Srinivasulu, 2012; Tsuruoka et al., 2020), the cell membrane model (Brosseau and Sabri, 2021), the RC network (Satish et al., 2020), the circuit structures (Hope et al., 2020; Mao et al., 2021), and the converter (Adapa et al., 2020; Liu et al., 2021b). The memristor is a nonlinear circuit device. Its nonlinear, synapse-like, and nonvolatile characteristics (Chua, 1971) are explored to emulate the dynamical activities of biological neurons. The flux-controlled memristor mimics the biological synapse, and the memristive Hindmarsh-Rose (HR) neuron can perform the complex dynamic behaviors of realistic neurons (Bao et al., 2020). The memristor bridge synapse used to build the multilayer neural network can solve the nonvolatile weight storage (Adhikari et al., 2012). Memristors are used as synapses to link the pre-neurons and post-neurons in the spiking networks (Hajiabadi and Shalchian, 2020). A memristive (Ag/SiO2/Au) integration-and-fire neuron can implement the primary functions of neurons (Hajiabadi and Shalchian, 2018). The deep stochastic spiking neural networks (SNN) are constructed by the memristive neuron and synapse crossbar, which fulfill the complex classification tasks with higher accuracy (Wijesinghe et al., 2013). The learning experience memristor (LEM) is employed to be a synapse in associative memory neural networks to simulate the biological neural networks (Zhang and Long, 2019). At the hardware level, a hardware circuit for columnar-organized memory is composed of the CMOS neuron, and the memristor crossbar arrays realize information storage and retrieval (Shamsi et al., 2018). Memristors and COMS transistors are combined to build the CMOS memristor structure, which successfully replicates the learning functions of biological neurons and the vital activities of the brain (Azghadi et al., 2017). The Pavlov associative memory circuit with a memristor can implement the essential functions of forgetting and learning (Wang and Wang, 2018). The sensor processing system with the self-healable materials and memristive switches can show the functions of the neuro-morphological nociceptors, local associative learning, and communication. It performs good fault tolerance and robustness (John et al., 2020). The MC circuits constructed by memristors and capacitors have also been widely studied and applied. The MC circuit experiments show the memristor does exist (Pershin and Ventra, 2018). The main application of the MC circuit is used as the chaos circuit (Wang et al., 2019; Deng and Li, 2020), filter circuits (Sozen and Cam, 2014; Gursul and Hamamci, 2019), relaxation oscillators (Mutlu, 2015), and charging circuit (John et al., 2016). Not only that, the MC circuit can be utilized to solve the differential equations (Fu et al., 2021), perform the complex dynamical behaviors (Chen et al., 2019), stochastic computing (Benito et al., 2021), and bursting oscillations (Njitacke et al., 2020). Meanwhile, memristors have obvious advantages in spiking neuron models and networks. A memristive neuron model can generate various firing patterns and firing multistability (Lin et al., 2020). The neuron dynamics can be stimulated by memristor circuits (Innocenti et al., 2019). Mott-memristor artificial neuron is realized and applied in the hardware implementation of SNN (Wei et al., 2021).

Despite the studies on the RC circuit and the memristor have been carried out widely, few studies have ever focused on the combination of the memristor and the RC circuit to simulate the firing behavior of biological neurons. Here, we introduce the memristor to the RC neuron circuit, and the MC neuron circuit is established. The charging and discharging processes in the RC and MC neuron circuits are compared and discussed in Section 2. In Section 3, the different stimuli are injected into the RC and MC neuron circuits, generating the action potentials under properly setting parameter values. The RC and MC neuron circuits exhibit asynchronous and synchronous behaviors of collective neurons in Section 4. To show the special advantage of the MC neuron circuit, we compare the five neuron circuits with the proposed one in Section 5. The conclusion of the paper is presented in Section 6.

## 2. The RC and MC Neuron Circuits

### 2.1. The RC Neuron Circuit

Two terminal elements of a resistor and a capacitor play different roles in the circuit network. The resistor will dissipate the energy produced in the circuit in the form of heat. The capacitor is different from the resistor. The capacitor characteristics appear in the circuit only when the stimulus variation happens. The capacitor is used as an energy storage component that cannot generate and dissipate energy. When the direct current passes, the capacitor acts as an open-circuit component. The capacitor becomes a short-circuit component when the alternating current passes. Therefore, the capacitor can pass the AC signals and block the DC signals. These characteristics make it become one of the significant elements in an electronic circuit to implement coupling, filtering, oscillating, phase shifting, and bypassing. The RC circuit (or the RC filter network) consists of a resistor and a capacitor. It is divided into the RC series and parallel circuits. In a parallel circuit, the potential difference between the two ends of each element is the same. When the direct current is injected into the circuit, the current only passes through a resistor. When the alternating current acts on the circuit, the total current equals the summation of resistive and capacitive currents. Another physical phenomenon: the capacitor plate connected to the positive pole of the power source lost electrons becomes positively charged, the capacitor plate attached to the negative side of the power source gained electrons becomes negatively charged. Both plates receive equal and opposite charges. The change of charges between plates causes the charge-discharge behavior of capacitors. The RC circuit is connected to the power supply, and the capacitor potential difference increases with time until it is equal to the power supply voltage. It performs the charging process of the RC circuit. We assume that the capacitor is fully charged in the RC circuit. The capacitor discharges through the resistor, and the capacitor potential difference decreases gradually with time until the discharge is completed and the capacitor potential becomes zero. It is the discharging process. The charging and discharging processes are affected by the values of the resistor and capacitor.

The electrical properties of the RC circuit are consistent with the physical properties of the cell membrane in neurons. Therefore, the studies of the RC circuit in neurobiology have been expanded dramatically. The parallel RC circuit can mimic the bilayer lipid membrane and performs membrane electrical properties (Ivanic and Tvarocek, 1998). The synapse cleft is simulated by the RC circuit, and the values of R and C is related to the transmission error rate (Taluk and Iik, 2020). Not only that, the RC circuit is used as the cell membrane model to evaluate the cell death rates (Fei and Xiao, 2006; Fei, 2018) and the voltage variation between normal and abnormal cells (Fei et al., 2009). At the same time, many spiking neuron models (Hodgkin and Huxley, 1952; FitzHugh, 1961; Nagumo et al., 1962; Morris and Lecar, 1981; Bernander et al., 1994; Izhikevich, 2003) are proposed based on the characteristics of the RC circuit. They reproduce the functional characteristics and behavior patterns in neurons from different levels. These research results of the RC circuit in neuroscience have proved the importance of realizing the characteristics of the cell membrane and mimicking the function of the cell membrane in neurons.

### 2.2. The MC Neuron Circuit

**Figure 1:**
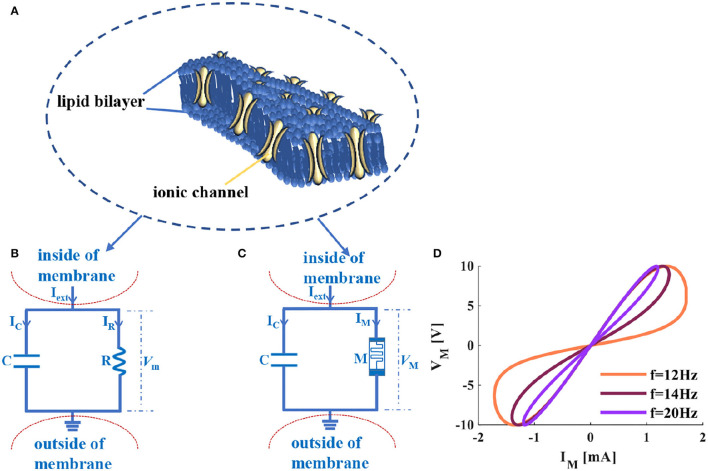
The cellular membrane of a neuron and its circuit models, **(A)** the diagram of the voltage-controlled ionic channel cell membrane, **(B)** the RC circuit of the neuron membrane, **(C)** the MC circuit of the neuron membrane, **(D)** the voltage-current relationship of a memristor.

Cellular membranes with complex and physical bilayer properties consist of proteins and lipids (Ballweg et al., 2020) (Figure 1A). The neuron cell membrane is an insulator that can separate the electrolytes fluid inside and outside the cell membrane (Tuma et al., 2016). In a neuron, currents can pass through the neuron membrane from inside to outside or from outside to inside. The cellular membrane of a neuron can be physically realized by the parallel circuit of a resistor and a capacitor (Figure 1B), which can be used to solve the event involving time, reproduce the neuron behavior, analyze the propagation mechanism and firing behaviors.

The resistor (*R* in Figure 1B) is equivalent to the ion channel of the cellular membrane of a neuron (the yellow regions of Figure 1A represent the voltage-controlled ion channels). Ionic channels selectively allow specific ions to pass through, and the opening and closing of the channel depend on the membrane potential. The capacitor (*C* in Figure 1B) corresponds to the phospholipid bilayer of the cellular membrane of a neuron (the dark blue area in Figure 1A) shows the phospholipid bilayer). The physical realization of the cellular membrane of a neuron is achieved, which is called the RC neuron circuit, as shown in Figure 1B. Real neurons in the resting state always maintain a constant voltage drop through the cellular membrane (the typical value is −65*mV*). The voltage outside the cellular membrane is usually defined as zero. The voltage inside the cellular membrane is related to external excitation (*I*_*ext*_ in Figure 1B). The voltage difference between inside and outside the cellular membrane is the membrane potential (*V*_*m*_ in Figure 1B) in the neuron. Therefore, the constructed RC circuit efficiently simulates the fundamental structure of the neuronal cell membrane.

The memristor is the nonlinear circuit element with the memory property (Ventra et al., 2009; Chen et al., 2020), which can be used to simulate the synapse (Kim et al., 2012), neuron (Chua et al., 2012b), and the Hodgkin-Huxley axon (Chua et al., 2012a; Chua, 2013). The ion channel of the neuron affects the signal propagation in the nervous system, and it has been proved that the memristor is an electronic analog device for mimicking the ion channel in the HH model (Feali and Ahmadi, 2017). It is mainly because the memristor shares the voltage-controlled characteristic with ion channels in neurons (Volkov et al., 2016). Therefore, the designed memristive Hodgkin-Huxley neuron model is realized by replacing the ion channel with the memristor (Hu and Liu, 2019) to present the dynamic properties in neurons.

According to the previous research results and the RC circuit model in Figure 1B, the memristor is introduced to replace the resistor. The parallel structure of a memristor and a capacitor is constructed, and the MC neuron circuit is obtained (Figure 1C). The flux-controlled memristor can be defined as (Wang et al., 2012):


(1) $$\begin{array}{l} M\left(\varphi \left(t\right)\right)=\{\begin{array}{cc} 20000 & \varphi \left(t\right)<-0.75\\ \sqrt{-3.98\times 10^{8}\varphi \left(t\right)+10^{8}}-0.75 & \leq \varphi \left(t\right)<0.25\\ 100 & \varphi (t))\geq 0.25 \end{array} \end{array}$$

The curve of a memristor distributes in the first and third quadrants, which performs the high-resistance and low-resistance states, and retains the memristance without the external power supply (Figure 1D). It behaves as the switching and nonvolatile characteristics of a memristor.

The external stimuli are applied to the RC (MC) neuron circuit (*I*_*ext*_ in Figures 1B,C) cause the generation of the ion concentration differences in extracellular and intracellular, resulting in the change in membrane potential (*V*_*m*_ and *V*_*M*_ in Figures 1B,C). According to biophysics, the circuit models (Figures 1B,C) realize the physical emulation of the cellular membrane of the neuron.

The following equations are obtained according to Kirchhoff's voltage-current law and the circuit model of Figures 1B,C.


(2) $$\begin{array}{l} I_{ext}=I_{C}+I_{R}\\ I_{ext}=I_{C}+I_{M} \end{array}$$

(3) $$\begin{array}{l} V_{C}=V_{R}=V_{m}\\ V_{C}=V_{M} \end{array}$$

(4) $$\begin{array}{l} dV_{m}/dt=I_{ext}/C-V_{m}/RC\\ dV_{M}/dt=I_{ext}/C-V_{M}/MC \end{array}$$

Where, *C* is the membrane capacitance, *R* is the membrane resistance, *M* is the memristor, *Iext* is the external stimulus, *I*_*C*_ is the current flowing through the membrane capacitor, *I*_*R*_ is the current flowing through the membrane resistor, *I*_*M*_ is the current flowing through the memristor, *V*_*m*_ and *V*_*M*_ are the membrane potentials of the RC and MC models, respectively. Equation (2) is the node current equation, (3) is the loop voltage equation, and (4) is the first-order differential equation.

## 3. The Comparison Between the RC and MC Neuron Circuits

To understand the electrical characteristics in the RC and MC neuron circuits, we discuss the mathematical simulations from three aspects: the charging process, the discharging process, and the generation of the action potential.

### 3.1. The Charging Processes of the RC and the MC Neuron Circuits

**Figure 2:**
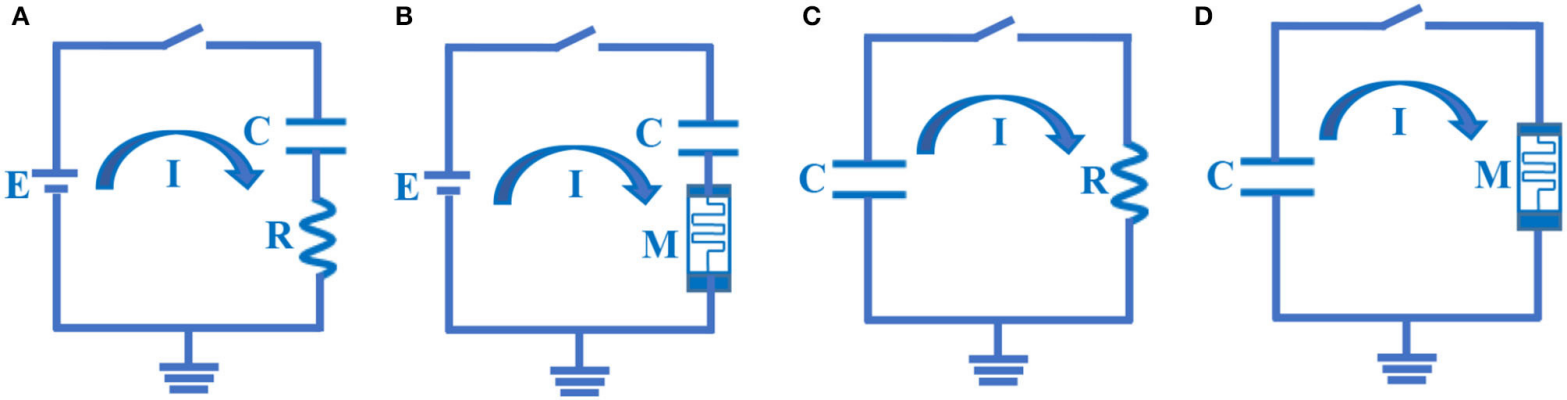
The RC and MC charging – discharging circuits. **(A)** The RC charging circuit. **(B)** The MC charging circuit. **(C)** The RC discharging circuit. **(D)** The MC discharging circuit.

The RC neuron circuit is so familiar to us. It is a first-order transient circuit, a simple structure with a few components. The signal transmission process in the RC circuit is similar to the behaviors of the neuron cell membrane. Therefore, it is widely studied as the simplest neuron circuit (Figure 2A).

Where, *E* is the battery power, *E* = 100*mV*; *C* is the capacitor, *C* = 10^−6^*F* is a typical value of neuron membrane capacitor; *R* is the resistor, *R* = 10^3^Ω is a specific value of neuron membrane resistor; τ is time constant, τ = *RC* = 10^−3^*s*. At time *t* = 0, the switch is on, the capacitor is initially uncharged in the series circuit, and the initial potential value of a capacitor is zero.

According to Kirchhoff's voltage-current law, the current passing through every element is identical in a series circuit *I* = *I*_*C*_ = *I*_*R*_. The power supply potential is equal to the sum of the potential difference between the two ends of each component: *E* = *V*_*C*_ + *V*_*R*_ (*VC* is the voltage at both ends of a capacitor, *V*_*R*_ is the voltage at both ends of a resistor). The relationship between the charge and the potential is *Q* = *CV*_*C*_. Thereby, The RC charging circuit can be described by the following equations:


(5) $$\begin{array}{l} V_{R}=RI_{R}=RCdV_{C}/dt=E-V_{C} \end{array}$$

(6) $$\begin{array}{l} V_{C}=E\left(1-e^{-t/\tau }\right) \end{array}$$

(7) $$\begin{array}{l} V_{R}=Ee^{-t/\tau } \end{array}$$

(8) $$\begin{array}{l} I=I_{C}=I_{R}=dq/dt=CdV_{C}/dt=Ee^{-t/\tau }/R \end{array}$$

The power equations of the electric source, the resistor, and the capacitor are as follows:


(9) $$\begin{array}{l} P_{E}=EI \end{array}$$

(10) $$\begin{array}{l} P_{C}=V_{C}I_{C} \end{array}$$

(11) $$\begin{array}{l} P_{R}=V_{R}I_{R} \end{array}$$

The first-order differential equation of the capacitor voltage can be defined as the finite difference form:


(12) $$\begin{array}{l} V_{C}\left(t+\Delta t\right)=V_{C}\left(t\right)-\Delta t/\tau \left(E-V_{C}\left(t\right)\right) \end{array}$$

**Figure 3:**
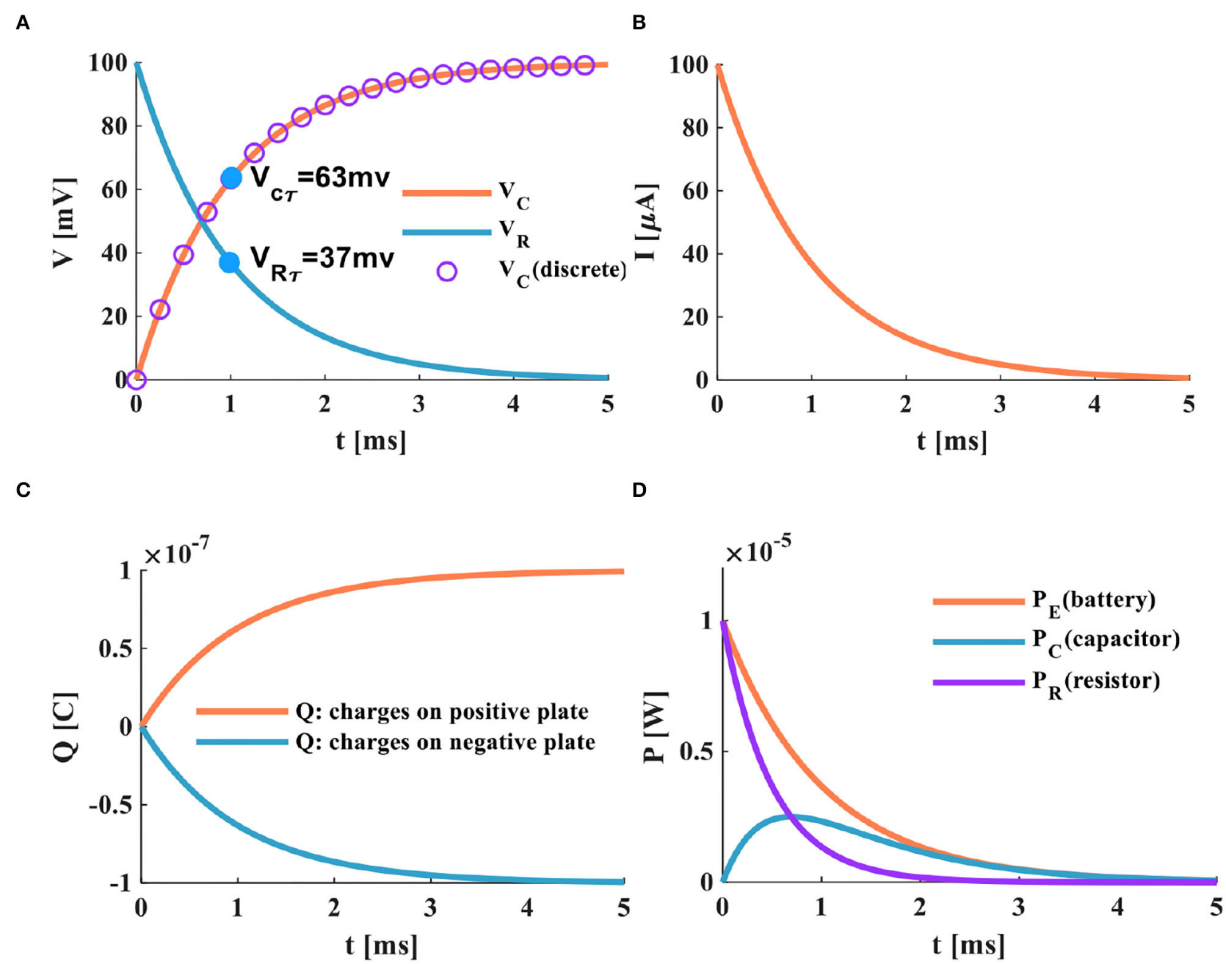
The charging process of the RC neuron circuit, **(A)** the charging process of the capacitor, **(B)** the charging current of the RC neuron circuit, **(C)** the changing of charges on positive and negative plates of the membrane capacitor, **(D)** the power variation of the RC neuron circuit.

Here, the initial time *t* = 0 and the initial potential is *V*_*C*_(0) = 0. Based on the above-given circuit equations and the relationship between current and voltage. When the switch is closed, the power supply charges to the capacitor through the resistor, the capacitor potential is initially zero, and the resistor potential equals the power source voltage. The plots of the charging process of the RC neuron circuit are shown in the following:

a) The switch is closed; the power supply charges the membrane capacitor. When τ = *RC* = 1*ms*, the membrane resistor potential decreases from *V*_*R*_ = 100 to *V*_*R*_ = 37*mV*, the membrane capacitor potential increases from *V*_*C*_ = 0 to *V*_*C*_ = 63*mV*. The total potential difference E is the sum of the capacitor potential *V*_*C*_ and the resistor potential *V*_*R*_. The curve represented by the circle is the discrete curve of the capacitor potential. The membrane capacitor potential increases gradually until the charging process is over, and the membrane resistance potential decreases until it tends to zero, as shown in Figure 3A.

b) When the switch is closed, the charges on the two plates of the membrane capacitor move in the direction to form the membrane current. During the charging process of the membrane capacitor, the membrane current is generated first, and then the membrane potential is produced. With the change of time, the charging process of the membrane capacitor is completed, the current in the circuit decreases exponentially to zero, as shown in Figure 3B.

c) In the RC circuit, the switch is closed, and the power supply charges the membrane capacitor through the membrane resistor. The charge on the two plates of the membrane capacitor increases exponentially. When the membrane capacitor is fully charged (*t* > 5τ), the membrane capacitor acts as an open switch in the DC circuit, and the circuit is in a disconnected state, as shown in Figure 3C.

d) At the initial time *t* = 0, the charge on the positive and negative plates starts to increase rapidly. At *t* = 5*ms*, the membrane capacitor is fully charged, the charging process is completed. Part of the power supply energy in the RC circuit is stored by the membrane capacitor and partly consumed by the membrane resistor. The power changes of the resistor, the capacitor, and the power supply are shown in Figure 3D.

The memristor is a nonlinear electronic device whose device resistance can be adjusted by maintaining the history of the external stimuli (Strukov et al., 2008; Joglekar and Wolf, 2009; Sheridan et al., 2017). Memristors have been applied to the emulation of synapses and neurons (Lu et al., 2020), the realization of neuromorphic computing (Wang et al., 2016), and the implementation of the bio-inspired intelligent system (Duan et al., 2020). Here, a memristor *M* is used to replace the membrane resistor *R* in Figure 2A to construct the novel circuit composed of a memristor and a membrane capacitor. The MC charging circuit model is achieved, as shown in Figure 2B.

Here, *E* is the battery power, *E* = 100*mV*; *C* is the capacitor, *C* = 10^−6^*F*; *M* is the memristor; τ is the time constant, τ = *MC*, which is transformed into a function of time. When *t* = 0, the switch is closed, and the MC series circuit is composed of a battery, a memristor, and a membrane capacitor. The membrane capacitor is not charged initially, and the initial potential of the membrane capacitor *V*_*C*_(0) = 0*mV*. *I*_*C*_ is the current flowing through the capacitor, *I*_*M*_ is the current flowing through the memristor, and *V*_*M*_ is the voltage at both ends of a memristor. The MC charging circuit can be described as:


(13) $$\begin{array}{l} \tau =MC \end{array}$$

(14) $$\begin{array}{l} V_{M}=MI_{M}=MCdV_{M}/dt \end{array}$$

(15) $$\begin{array}{l} I=I_{C}=I_{M}=Ee^{-t/\tau }/M \end{array}$$

**Figure 4:**
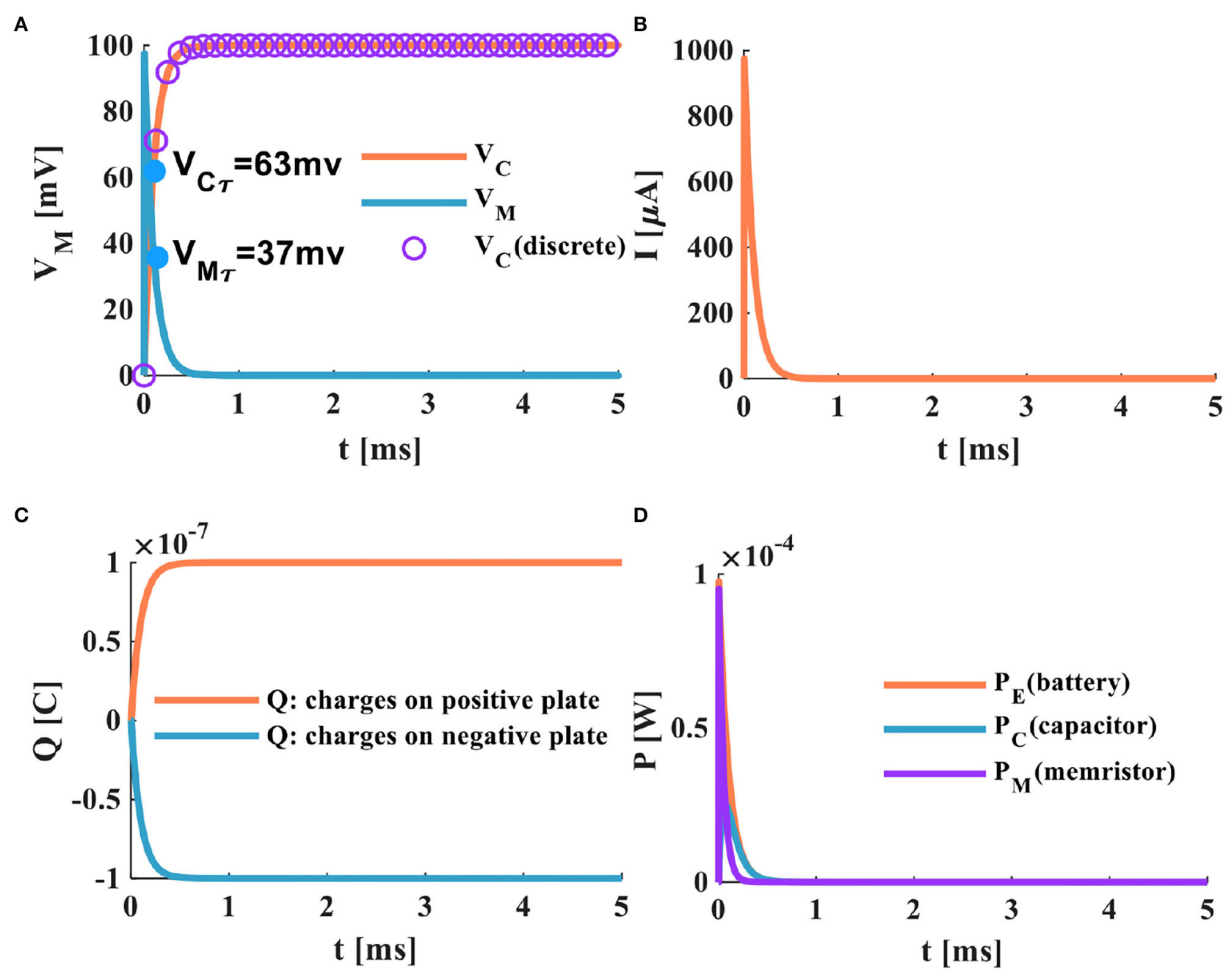
The charging process of the MC neuron circuit, **(A)** the membrane capacitor charging process, **(B)** the charging current of the MC neuron circuit, **(C)** the changing of charges on positive and negative plates of the membrane capacitor, **(D)** the power variation of the MC neuron circuit.

**Figure 5:**
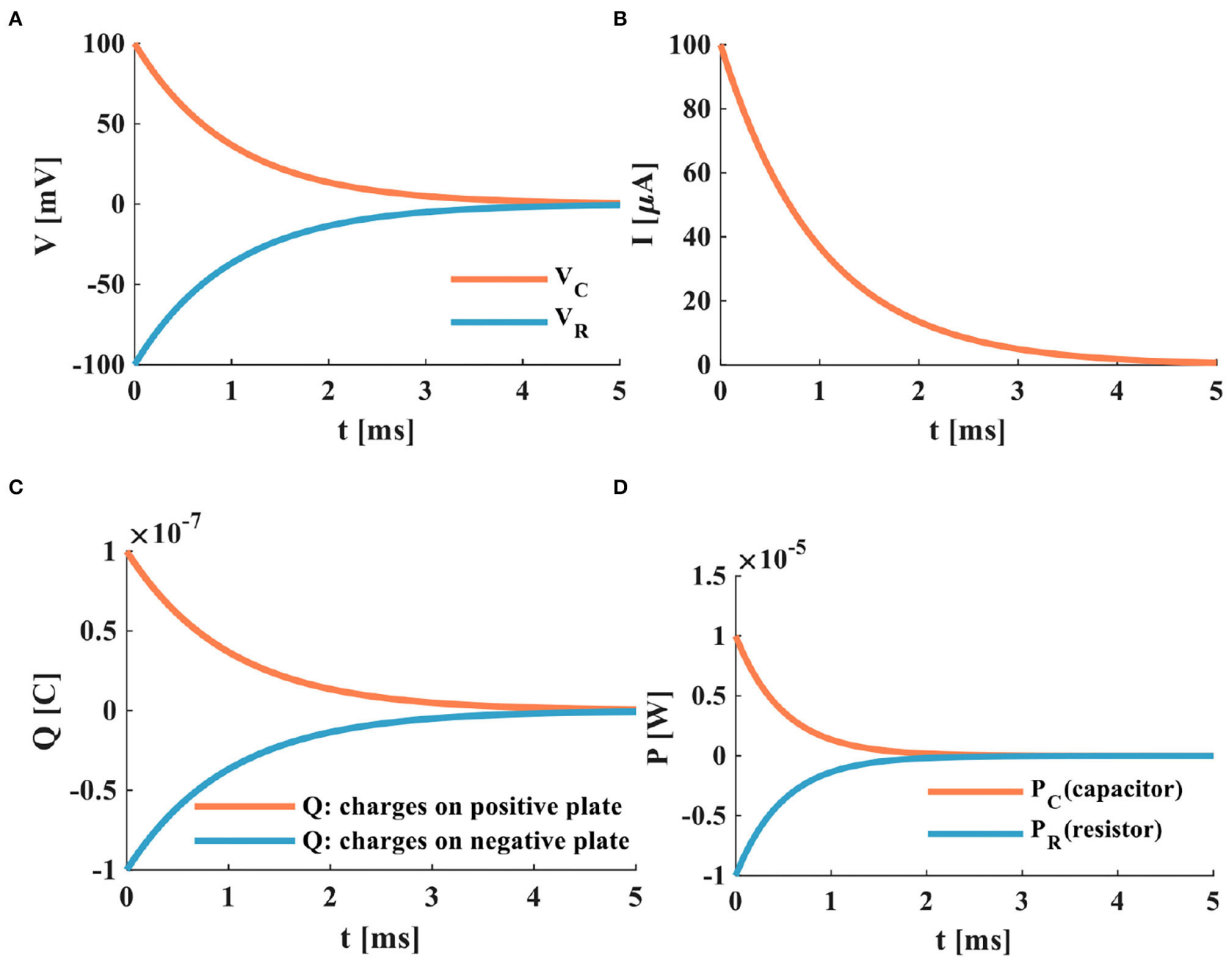
The discharging process of the RC neuron circuit, **(A)** the membrane capacitor discharging process, **(B)** the discharging current of the RC neuron circuit, **(C)** the charges on positive and negative plates of the membrane capacitor, **(D)** the power variation of the RC neuron circuit.

According to the current-voltage relationship mentioned above, the power supply charges the membrane capacitor through the memristor when the switch is closed. The membrane capacitor potential is zero at the initial time (*t* = 0*ms*); therefore, the memristor potential is equal to the power supply voltage. The charging process of the MC circuit is as follows:

a) When the switch is closed, the power supply of the MC circuit begins to charge the membrane capacitor through the memristor. When τ = 0.1*ms*, the membrane capacitor potential increases to 63%, *V*_*Cτ*_ = 63*mV*, and the memristor potential decreases to 63%, *V*_*Mτ*_ = 37*mV* (Figure 4A). The time constant is the characterization of the charge-discharge speed. The smaller τ, the shorter the charge-discharge time of the membrane capacitor, and the faster the charge-discharge speed. Therefore, the time constant of the MC circuit is much smaller than that of the RC circuit. It means that the charging speed of the MC circuit is faster than that of the RC circuit.

b) When the switch is in the closed state, the current generated by the MC circuit decreases exponentially with the end of the charging of the membrane capacitor. We compare the MC circuit with the RC circuit. When the time is about 0.5*ms*, the charging of the capacitor ends, and the MC circuit current tends to zero (Figure 4B). When the time is about 4*ms*, the charging of the capacitor is over, and the RC circuit current returns to zero (Figure 3B). The current generated by the MC circuit (Figure 5B) decreases faster than that produced by the RC circuit (Figure 3B).

c) After the switch is closed at about 0.5*ms*, the membrane capacitor of the MC circuit is fully charged. The charges on the positive and negative plates reach the maximum, as shown in Figure 4C.

d) The charges of the capacitor plates quickly accumulate to a peak in the MC circuit, the energy stored by the membrane capacitor is dissipated through the memristor, and the variation of the power is shown in Figure 4D.

### 3.2. The Discharging Processes of the RC and MC Neuron Circuits

The membrane resistor and capacitor are connected in parallel to form the RC discharge circuit model. Here, the charged membrane capacitor *C*, when *t* = 0, the switch is closed, the membrane capacitor discharges through the membrane resistor, and the RC discharging circuit is shown in Figure 2C.

Here, *C* represents the charged membrane capacitor, *C* = 10^−6^*F*, R denotes the neuron membrane resistor, *R* = 10^3^Ω, and τ is the time constant.

According to Kirchhoff's voltage-current law, the mathematical expressions of the RC discharging circuit are described as follows.


(16) $$\begin{array}{l} V_{R}=-V_{C} \end{array}$$

(17) $$\begin{array}{l} V_{C}=Q/C \end{array}$$

The initial time *t* = 0*ms*, *V*_*C*0_ = 100*mV*, and *Q*_0_ = *CV*_*C*0_ (*V*_*C*0_ is the initial potential of the membrane capacitor, *Q*_0_ is the initial charge, *V*_*R*_ is the voltage at both ends of a resistor, and *V*_*C*_ is the voltage at both ends of a capacitor).


(18) $$\begin{array}{l} V_{R}=RdQ/dt \end{array}$$

(19) $$\begin{array}{l} Q=Q_{0}e^{-t/\tau } \end{array}$$

(20) $$\begin{array}{l} V_{R}=-V_{C0}e^{-t/\tau } \end{array}$$

(21) $$\begin{array}{l} V_{C}=V_{C0}e^{-t/\tau } \end{array}$$

(22) $$\begin{array}{l} I=I_{C}=I_{R}=-V_{C0}/Re^{-t/\tau } \end{array}$$

When the switch is closed, the charged membrane capacitor begins to discharge through the resistor. The discharge process of the RC neuron circuit is shown in Figure 5.

With the increase of time, the potential of the membrane capacitor decreases exponentially from 100 to 0*mV*, and the membrane resistor potential changes from −100 to 0*mV*, and the time-consuming is 5*ms*. The sum of the potentials of the membrane capacitor and the membrane resistor is zero. When τ = 1*ms*, the membrane capacitor potential is 63*mV*, and the membrane resistor potential is −63*mV* (Figure 5A). The current generated in the RC neuron circuit decreases exponentially. When τ = 1*ms*, the loop current decreases by 63% (Figure 5B). The charged membrane capacitor discharges exponentially. When the discharge process is complete, the charges of the two plates are zero (Figure 5C). The membrane capacitor discharges through the membrane resistor, and the powers of the membrane capacitor and the membrane resistor attenuate to zero at about 2.7*ms* (Figure 5D).

In the following, a resistor is replaced in the RC discharging circuit (Figure 2C) with a memristor, and the MC discharging circuit is constructed, as shown in Figure 2D.

According to Kirchhoff's voltage-current law, the MC discharging circuit is defined as:


(23) $$\begin{array}{l} V_{M}=MdQ/dt \end{array}$$

(24) $$\begin{array}{l} V_{M}=-V_{C0}e^{-t/\tau } \end{array}$$

(25) $$\begin{array}{l} I=I_{C}=I_{M}=-V_{C0}/Me^{-t/\tau } \end{array}$$

The *C* represents the charged membrane capacitor, *C* = 10^−6^*F*, M denotes the memristor, *V*_*C*0_ = 100*mV* is the initial potential of the membrane capacitor, *V*_*M*_ is the voltage at both ends of a memristor, and *V*_*C*_ is the voltage at both ends of a capacitor.

**Figure 6:**
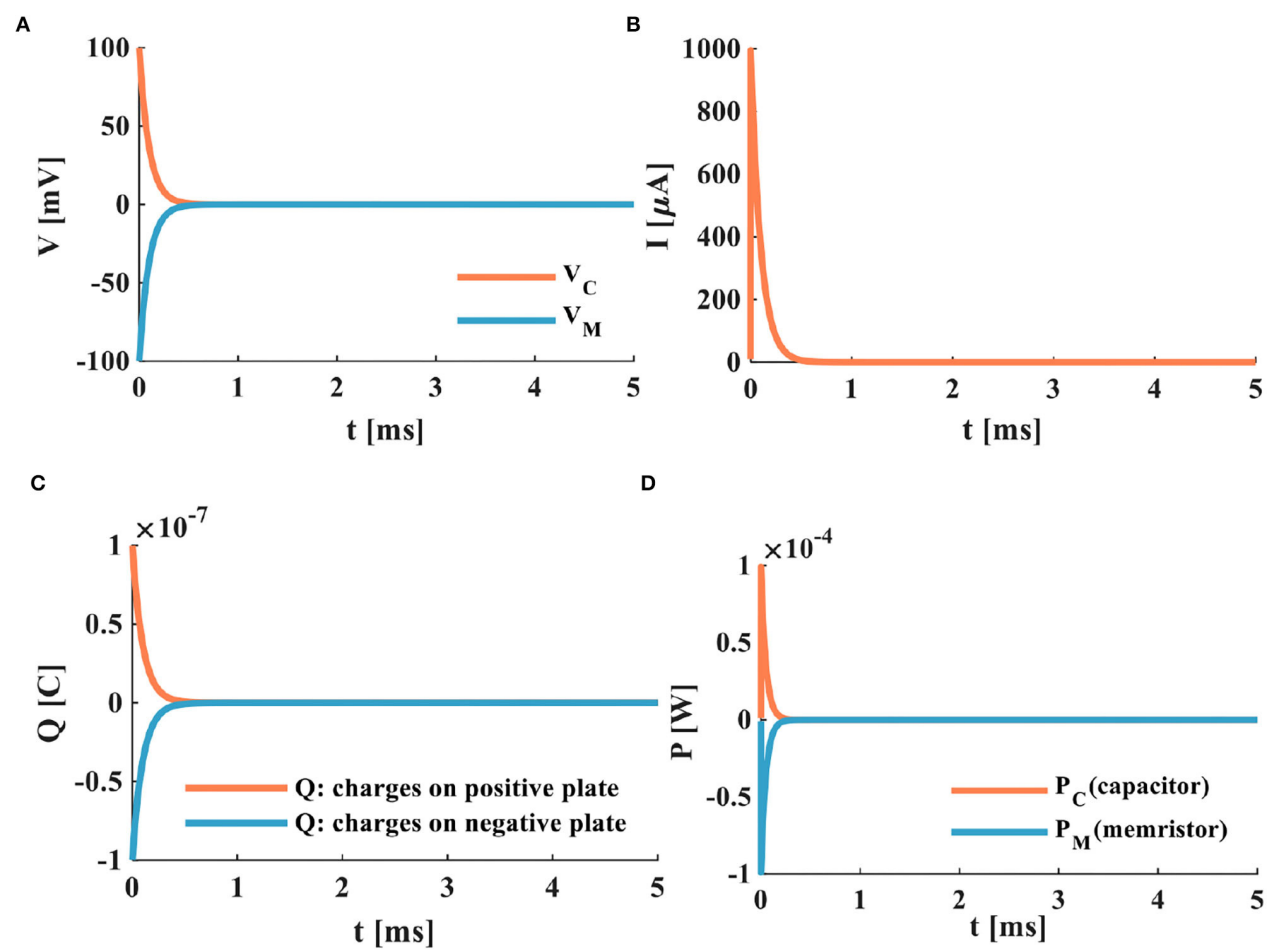
The discharging process of the MC neuron circuit, **(A)** the membrane capacitor discharging process, **(B)** the discharging current of the MC neuron circuit, **(C)** the discharging of charges on positive and negative plates of the membrane capacitor, **(D)** the power variation of the MC neuron circuit.

When the switch of the MC discharging circuit is closed, the fully charged membrane capacitor discharges through the memristor. The discharging process of the MC neuron circuit is shown in Figure 6.

Comparing the RC neuron circuit with the MC neuron circuit, the discharging speed of the MC neuron circuit is faster than that of the RC neuron circuit (the complete discharge of the RC neuron circuit needs about 4*ms* (Figure 5A), and the total discharging time of the MC neuron circuit only requires about 0.5*ms* (Figure 6A). In Figure 6A, the discharge current of the MC neuron circuit decreases exponentially through the memristor, and the current attenuates to zero after about 0.5*ms*. In Figure 5B, the current of the RC neuron circuit takes more than 5*ms* to decay to zero. Therefore, the discharging current of the MC neuron circuit attenuates faster than that of the RC neuron circuit. The charges on the positive and negative plates of the membrane capacitor in the MC neuron circuit decrease exponentially, and the discharge is completed needs about 0.5*ms* (Figure 6C). However, the RC neuron circuit takes about 4*ms* to complete the discharge (Figure 5C). The capacitor and memristor power require about 0.3*ms* to decrease to zero (Figure 6D). However, the RC neuron circuit takes about 2.3*ms* to consume the energy (Figure 5D). The MC neuron circuit has a faster charging and discharging speed than the RC neuron circuit.

### 3.3. The Different External Stimuli Are Applied to the RC and the MC Neuron Circuits

The membrane potentials are ubiquitous biophysical phenomena throughout the neural system, which describe the spatiotemporal patterns and dynamic behaviors of electrical signals (Xu et al., 2017; Liu et al., 2021a). The external stimulus affects the generation of the membrane potential of neurons. Various stimulus forms and different stimulus intensities are directly related to the firing patterns of neurons. We apply the step current, a series of pulse currents, and the single pulse current to the RC and MC neuron circuits. Here, the capacitance value *C* = 10^−6^*F*, the resistance value in the RC neuron circuit *R* = 10, 000Ω, the memristance value in the MC neuron circuit varies from 20,000 to 100 Ω, and its initial value *M*(0) = 10, 000Ω. According to (4), we get the following simulation results.

### 3.4. The External Stimulus Is the Step Current

The step current (*I*_*ext*_ = 10μ*A*, its action time is 30*ms*) is applied to the RC neuron circuit (Figure 1B) and the MC neuron circuit (Figure 1C), and the related parameters of the two circuits are consistent. The time constant in the RC neuron circuit depends on the membrane resistance and the membrane capacitance. The time constant of the MC neuron circuit is a function of time, which is different from that of the RC neuron circuit.

**Figure 7:**
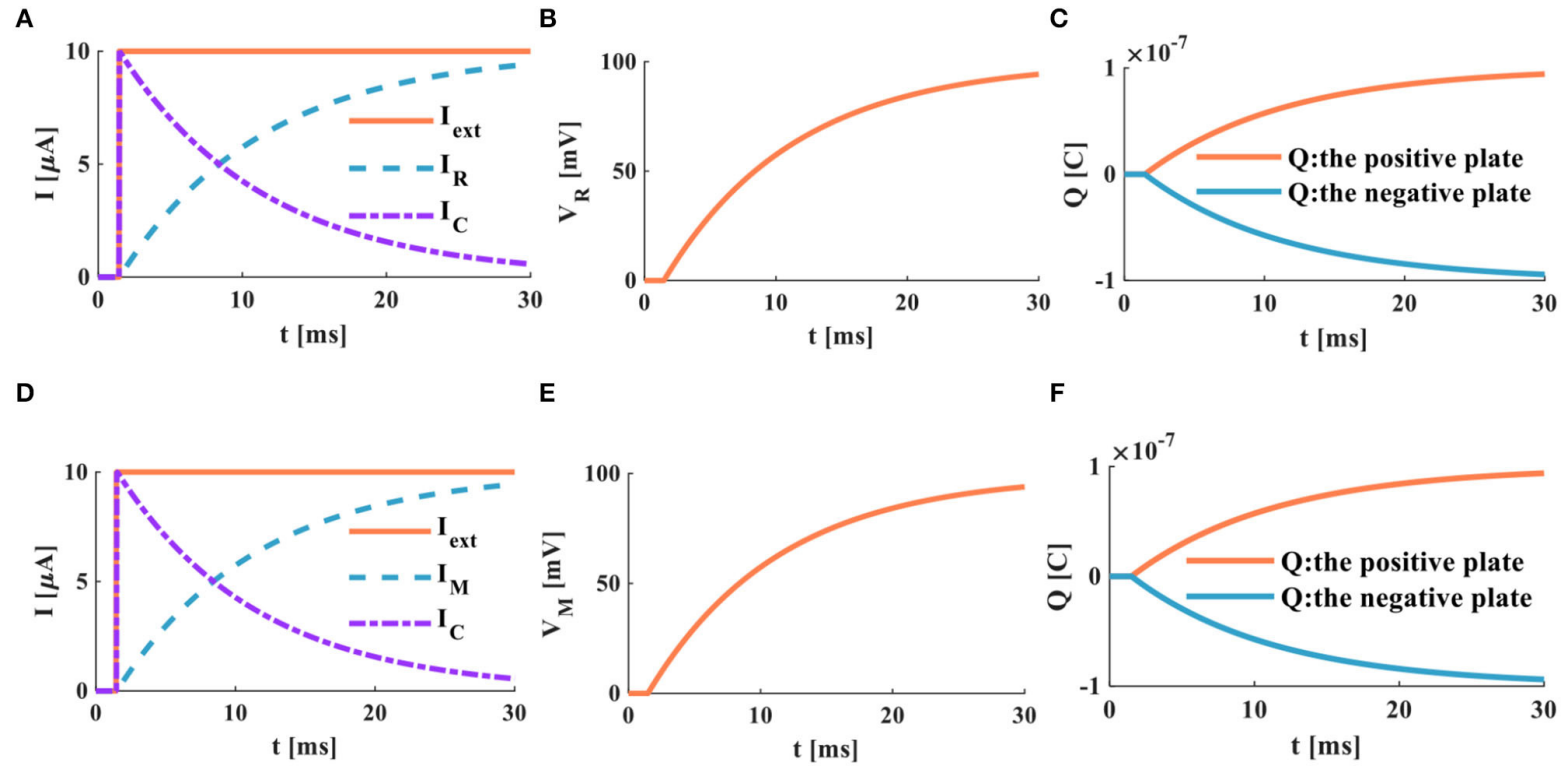
The step current acts on the RC and MC neuron circuits, **(A)** the current flows through the resistor R and the capacitor C, **(B)** the membrane potential of the RC circuit, **(C)** the charges on the positive and negative plates in the RC circuit, **(D)** the current flows through the memristor M and the capacitor C, **(E)** the membrane potential of the MC circuit, **(F)** the charges on the positive and negative plates in the MC circuit.

The RC and MC neuron circuits show the same waveforms under the action of the step current. There is no external stimulus in the RC neuron circuit from 0 to 3*ms*, and the membrane capacitor current and the membrane resistor current are zero. When the external current jumps to 10μ*A*, the membrane capacitor is instantly charged and dissipates energy through the membrane resistance. When *I*_*R*_ = *I*_*C*_ = *I*_*ext*_/2, it takes 8.392*ms*. With the increase of time, the membrane capacitor current attenuates exponentially, and the membrane resistor current increases exponentially (Figure 7A). The membrane potential of the RC neuron circuit increases exponentially, and the waveform of the membrane resistor current is the same as that of membrane potential (Figure 7B). The charge on the positive and negative plates of the membrane capacitor increases exponentially (Figure 7C). When the external current is applied to the MC neuron circuit, the membrane capacitor is charged instantly. With the increase of time, the membrane capacitance current and membrane memristor current change exponentially. When *I*_*M*_ = *I*_*C*_ = *I*_*ext*_/2, it takes 8.286*ms* (Figure 7D). The membrane potential of the MC neuron circuit increased exponentially (Figure 7E). The charges on the positive and negative plates of the membrane capacitor rise exponentially (Figure 7F). The simulation results indicate that the discharge speed of the MC neuron model is faster than that of the RC neuron model.

### 3.5. The External Stimuli Are a Series of Pulses

Neurons in the nervous system always receive electrical signals from other neurons, which means that the total electrical signal equals the sum of all the electrical signals received by neurons. Therefore, when a series of signals act on the neurons, the degree of depolarization in neurons increases, and the firing behavior occurs with the continuous accumulation of signals. The action time of the external stimulus affects the depolarization degree of the action potential. When the action time is short, the depolarization degree of the action potential is diminutive. When the action time is long enough, the depolarization degree of the action potential is significant.

The external stimulus consists of 16 continuous current pulses (*I*_*ext*_ = 10μ*A*) with an action time of 100*ms*, resulting in a small depolarized action potential.

**Figure 8:**
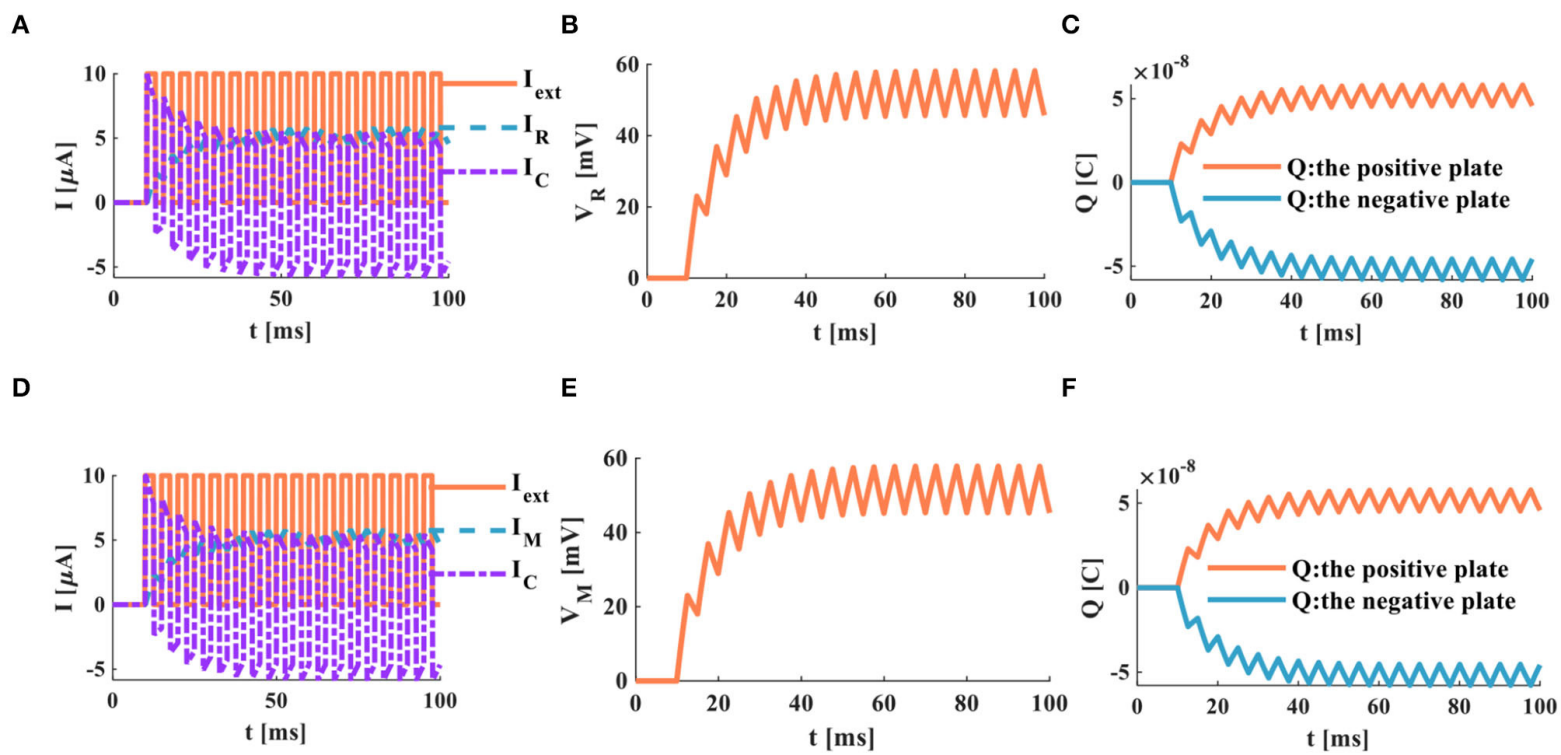
A series of current pulses act on the RC and MC neuron circuits, **(A)** the current flows through the resistor R and the capacitor C, **(B)** the membrane potential of the RC circuit, **(C)** the charges on the positive and negative plates in the RC circuit, **(D)** the current flows through the memristor M and the capacitor C. **(E)** the membrane potential of the MC circuit, **(F)** the charges on the positive and negative plates in the MC circuit.

A series of current pulses are applied to the RC neuron circuit, and the membrane capacitance shows obvious charging and discharging behaviors. The membrane resistor produces a small oscillating current (Figure 8A), whose waveform is consistent with the membrane potential waveform (Figure 8B). The charges on the positive and negative plates of the membrane capacitor show a small oscillation waveform (Figure 8C). When the MC neuron circuit receives the external stimulus, the membrane capacitor repeatedly performs the charging and discharging behaviors. The membrane memristor produces a small oscillating current (Figure 8D). The membrane potential performs the small oscillating behavior (Figure 8E). The charges on the positive and negative plates of the membrane capacitor are also a tiny oscillation waveform (Figure 8F). The MC and RC neuron models generate small oscillatory action potentials caused by the short charging time of the membrane capacitor.

**Figure 9:**
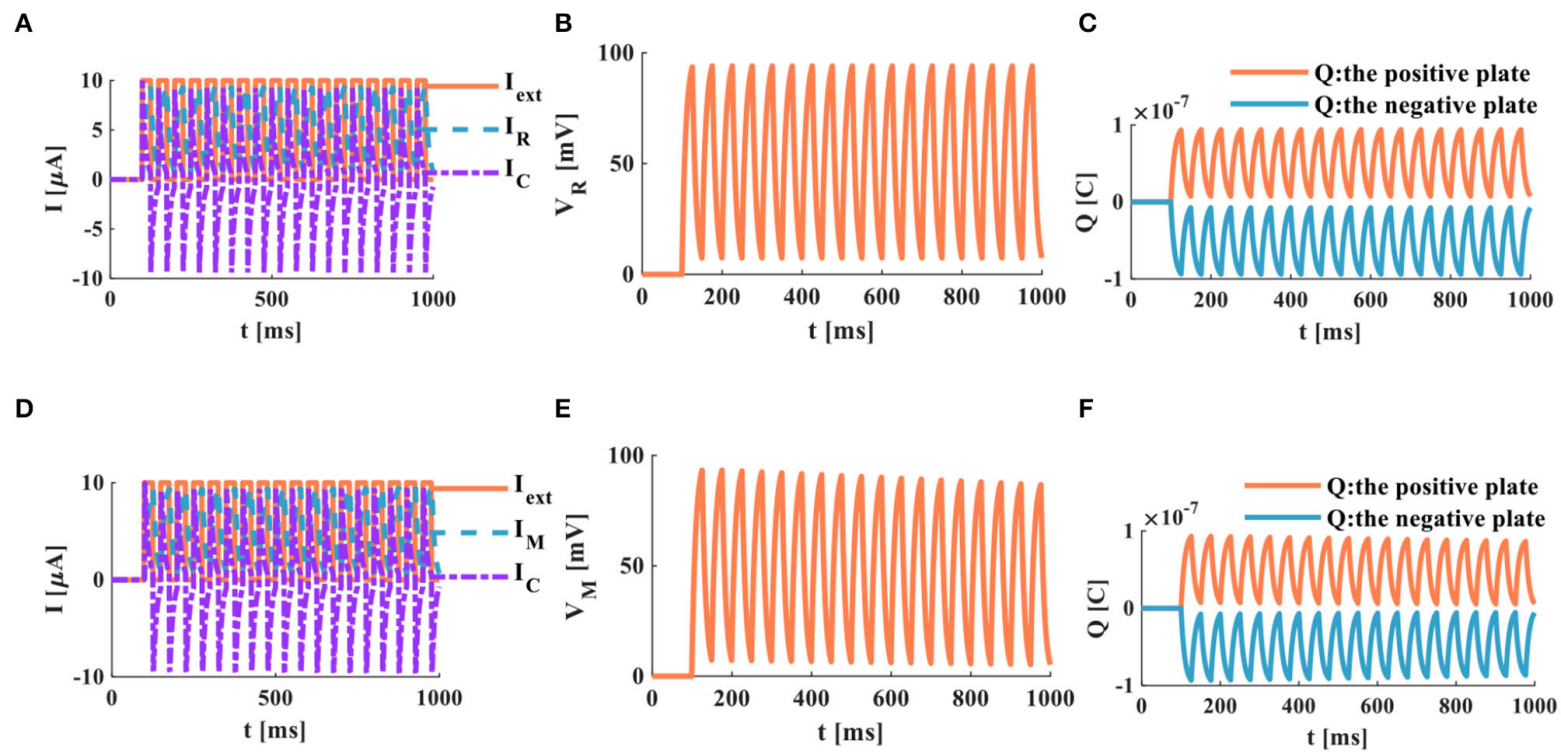
A series of current pulses act on the RC and the MC neuron circuits (the number of external stimuli is 16), **(A)** the current flows through the resistor R and the capacitor C, **(B)** the membrane potential of the RC circuit, **(C)** the charges on the positive and negative plates in the RC circuit, **(D)** the current flows through the memristor M and the capacitor C, **(E)** the membrane potential of the MC circuit, **(F)** the charges on the positive and negative plates in the MC circuit.

When the neuron receives a series of continuous current pulses, the membrane capacitor is given enough time to charge and discharge, and action potentials with more significant depolarization are generated (Figures 9B,E). In this way, the action potential can be propagated along the neuron axon and transmitted to other neurons.

In the RC neuron circuit, the action time of the external stimulus is extended to 1, 000*ms*. The membrane capacitor current exhibits an obvious charge-discharge process, and the membrane resistor current oscillates significantly (Figure 9A). The RC neuron circuit generates periodically oscillating action potentials (Figure 9B). The charges on the positive and negative plates of the membrane capacitor vary in the form of periodic oscillation (Figure 9C). The current of the membrane capacitor in the MC neuron circuit performs the repetitive oscillation behaviors (Figure 9D). The action potential waveform generated by the MC neuron circuit is consistent with the membrane resistance current waveform with the periodic oscillation (Figure 9E). The charges on the positive and negative plates of the membrane capacitor show the periodic oscillation phenomenon (Figure 9F). The RC and MC neuron models can realize the generation of the action potentials, which provides an effective way to mimic the behaviors of biological neurons.

**Figure 10:**
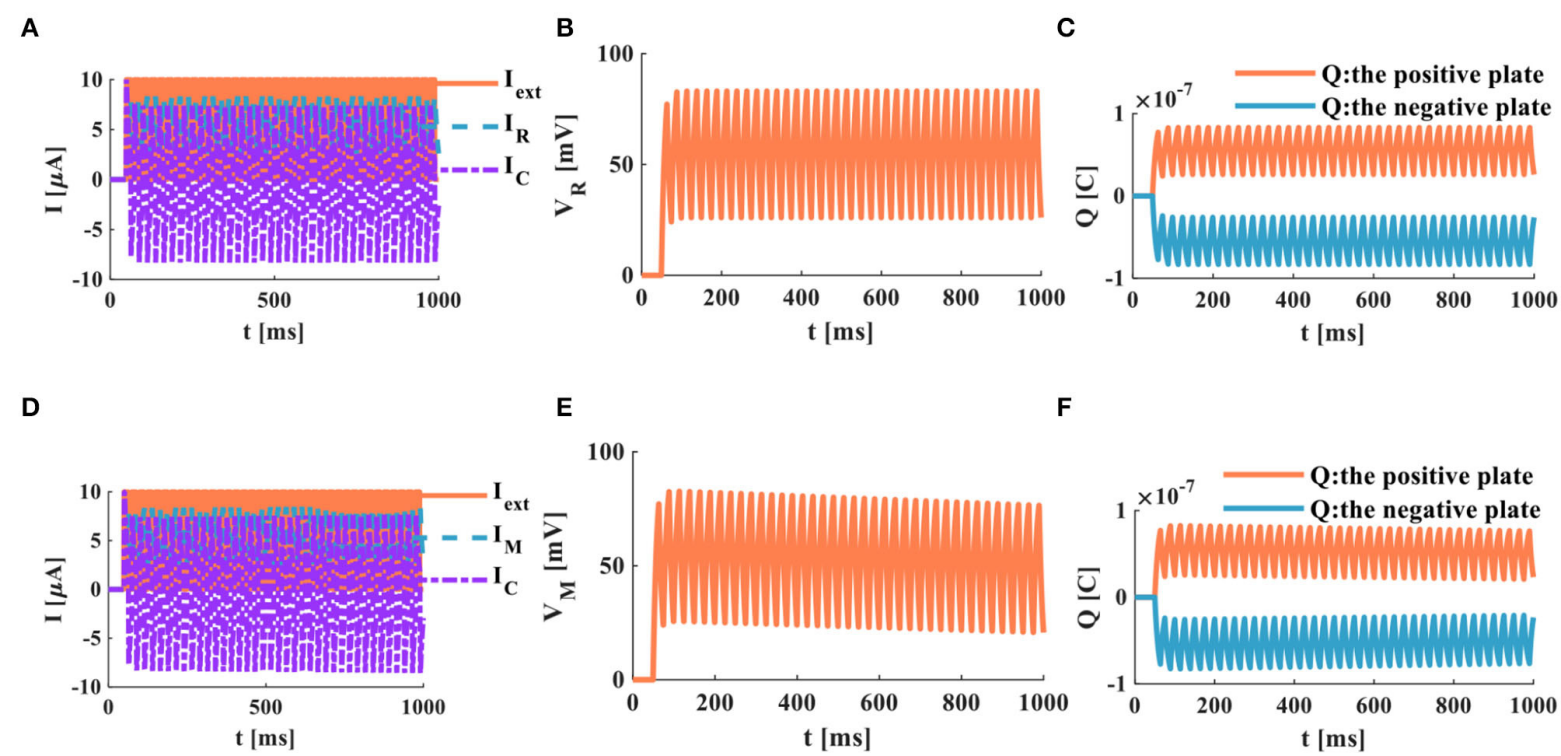
A series of current pulses act on the RC and MC neuron circuits (the number of external stimuli is 38), **(A)** the current flows through the resistor R and the capacitor C, **(B)** the membrane potential of the RC circuit, **(C)** the charges on the positive and negative plates in the RC circuit, **(D)** the current flows through the memristor M and the capacitor C, **(E)** the membrane potential of the MC circuit, **(F)** the charges on the positive and negative plates in the MC circuit.

The number of external stimulus pulses is increased to 38, and the action time is 1, 000*ms*. The RC and the MC neuron circuits are affected by the external stimulus (Figures 10A,D) produce a series of periodically oscillating action potentials (Figures 10B,E). Comparing Figures 9B,E with Figures 10B,E, the more the number of external stimuli, the more action potentials are generated. Meanwhile, a lot of charges are accumulated on the positive and negative plates of the membrane capacitor (Figures 10C,F). The more the number of external stimulus pulses, the more action potentials are generated.

**Figure 11:**
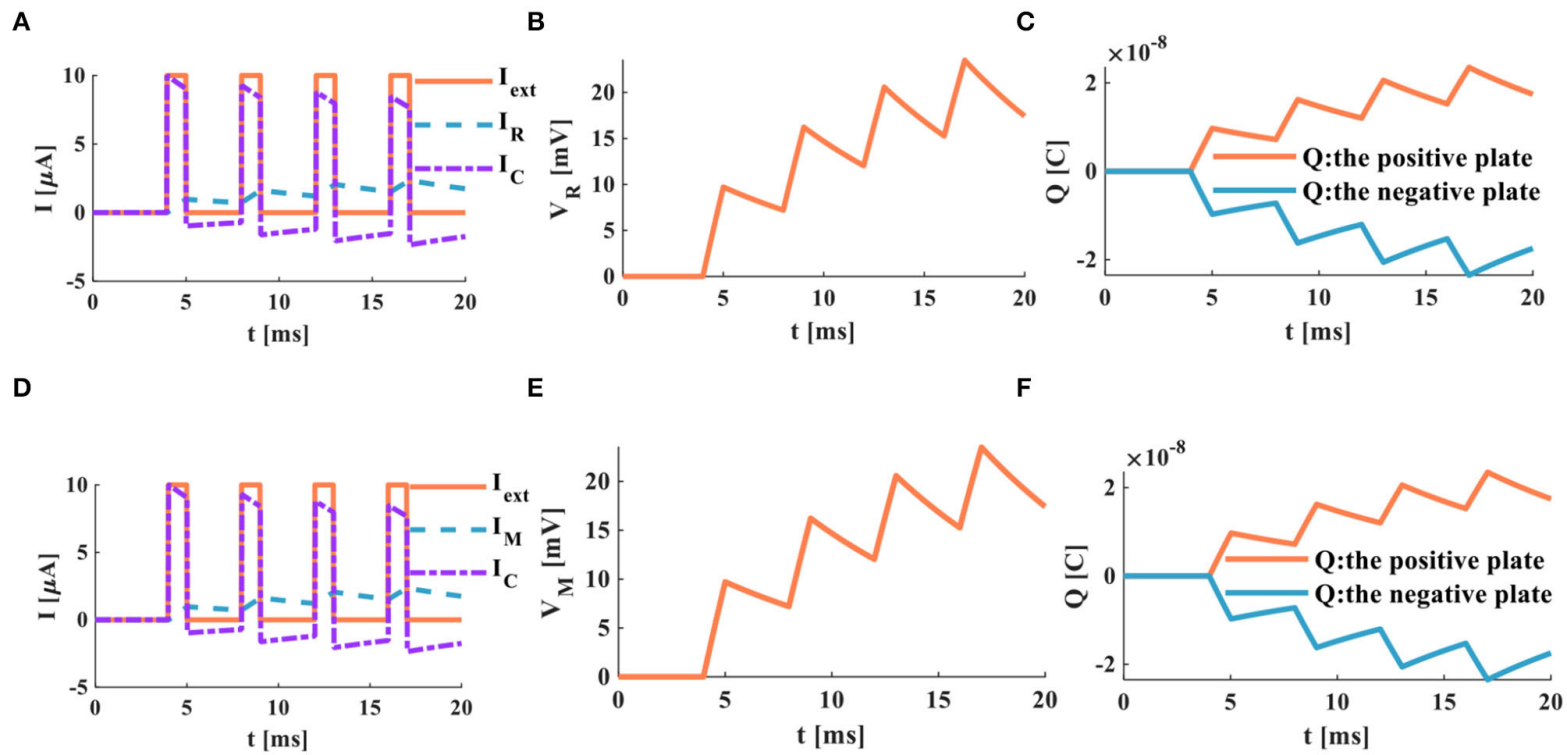
A series of current pulses act on the RC and MC neuron circuits (the number of external stimuli is 4), **(A)** the current flows through the resistor R and the capacitor C, **(B)** the membrane potential of the RC circuit, **(C)** the charges on the positive and negative plates in the RC circuit, **(D)** the current flows through the memristor M and the capacitor C, **(E)** the membrane potential of the MC circuit, **(F)** the charges on the positive and negative plates in the MC circuit.

When the charge and discharge time of the RC and MC neuron circuits is insufficient, in this case, the two circuits perform the superposition process of the membrane potential, and no action potential is generated. Four continuous pulses (*I*_*ext*_ = 10μ*A*) are used as the external stimulus to act on the RC and MC neuron circuits, and the action time is 20*ms* (Figure 11).

The charge and discharge phenomenon of the membrane capacitor in the RC circuit is not apparent. The membrane resistor current shows the superposition behavior (Figure 11A). The membrane potential generated by the RC circuit is the same as the membrane resistor current, and no action potential is generated (Figure 11B). The charges on the positive and negative plates of the membrane capacitor exhibit a superposition process (Figure 11C). The membrane capacitor in the MC circuit does not generate an evident charge-discharge phenomenon. The memristor current performs the superposition behavior (Figure 11D). The membrane potential and the charges on the positive and negative plates of the membrane capacitor only show the superposition behavior (Figures 11E,F). According to the above simulation results, when the action time of the external stimulus is short, the RC and MC circuits cannot produce the action potential.

**Figure 12:**
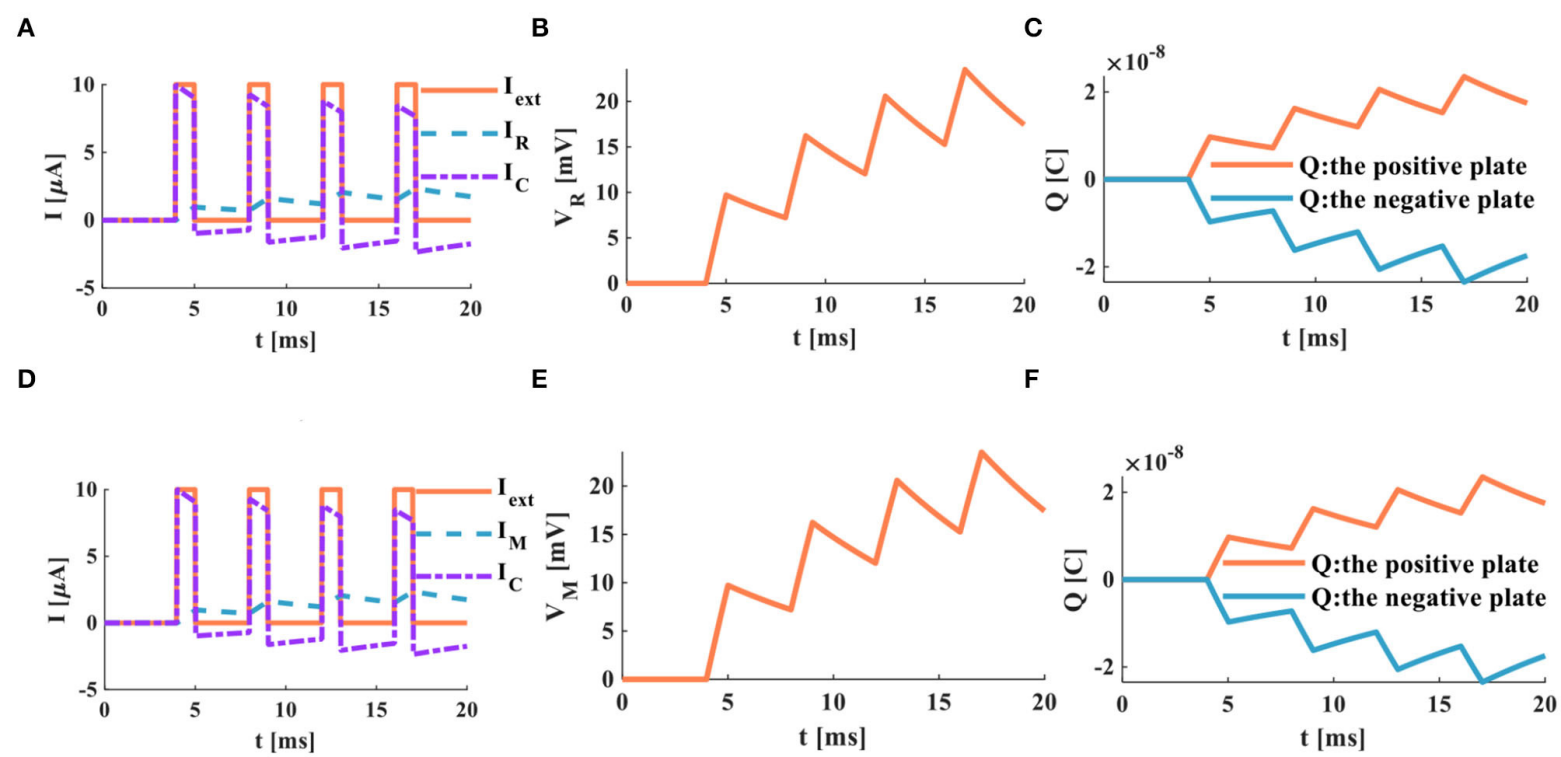
A series of current pulses act on the RC and the MC neuron circuits (the number of the external stimuli is 4, the action time is 1, 000*ms*), **(A)** the current flows through the resistor R and the capacitor C, **(B)** the membrane potential of the RC circuit, **(C)** the charges on the positive and negative plates in the RC circuit, **(D)** the current flows through the memristor M and the capacitor C, **(E)** the membrane potential of the MC circuit, **(F)** the charges on the positive and negative plates in the MC circuit.

The action time of the external stimulus is extended from 20 to 1, 000*ms*, and the external stimulus *Iext* = 10μ*A* (Figure 12).

When the RC and MC neuron circuits are given enough time to charge and discharge the capacitor, the action potentials are produced. The action time of the external stimulus affects the generation of the action potential.

### 3.6. The External Stimulus Is a Single Pulse

An individual pulse acts on the RC and MC neuron circuits, and the action time is 20*ms*, *I*_*ext*_ = 10μ*A*.

**Figure 13:**
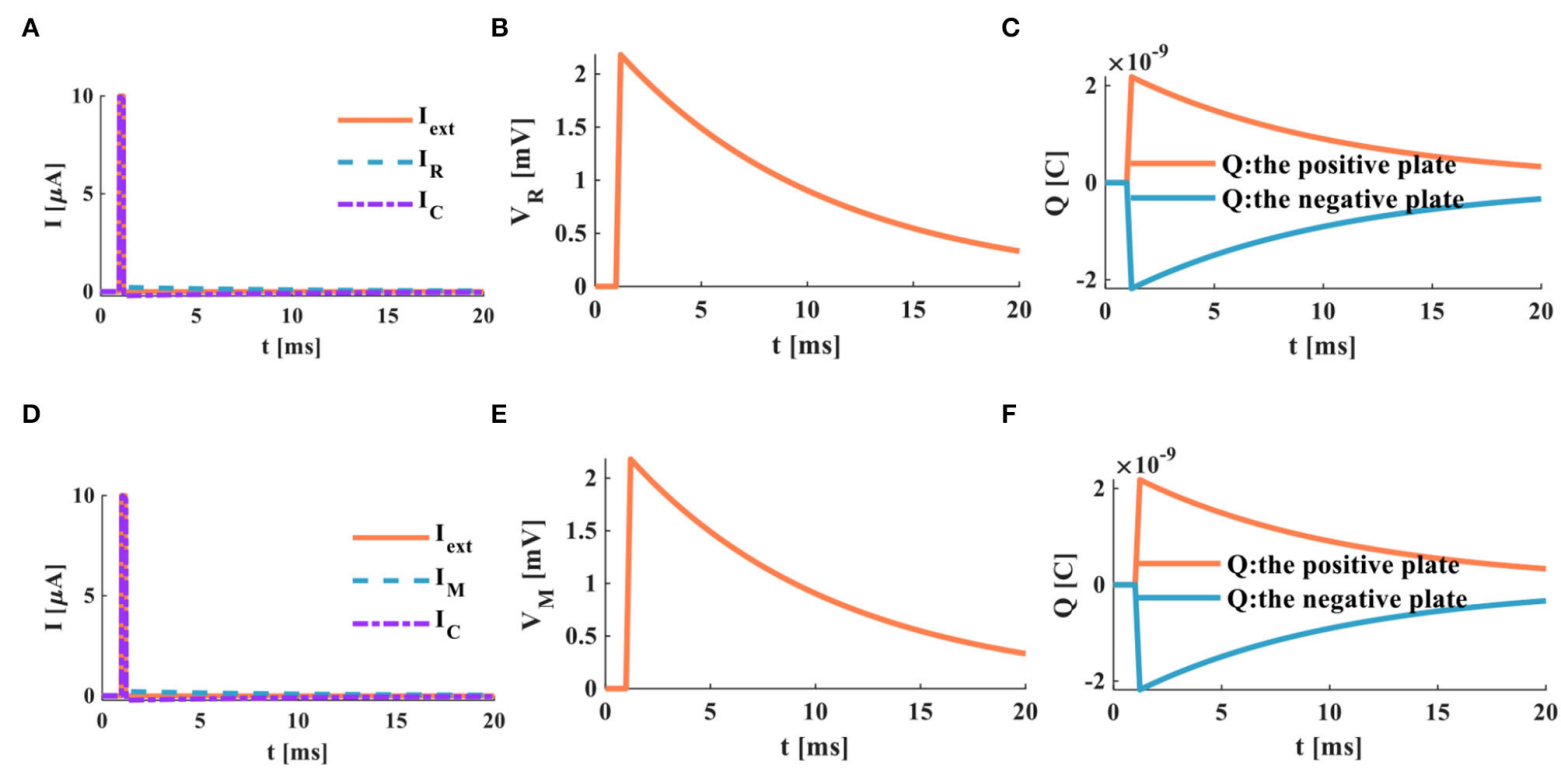
A single pulse acts on the RC and MC neuron circuits, **(A)** the current flows through the resistor R and the capacitor C, **(B)** the membrane potential of the RC circuit, **(C)** the charges on the positive and negative plates in the RC circuit, **(D)** the current flows through the memristor M and the capacitor C, **(E)** the membrane potential of the MC circuit, **(F)** the charges on the positive and negative plates in the MC circuit.

When the single pulse arrives, the RC and MC neuron circuits cannot generate the action potentials (Figures 13B,E). The membrane capacitor and the membrane resistor produce the minimal instantaneous current (Figures 13A,D). The two plates of the membrane capacitor are charged and discharged in 20*ms* (Figures 13C,F).

When the action time of the external stimulus is extended to 1000*ms*, the RC and MC neuron circuits generate the action potentials.

**Figure 14:**
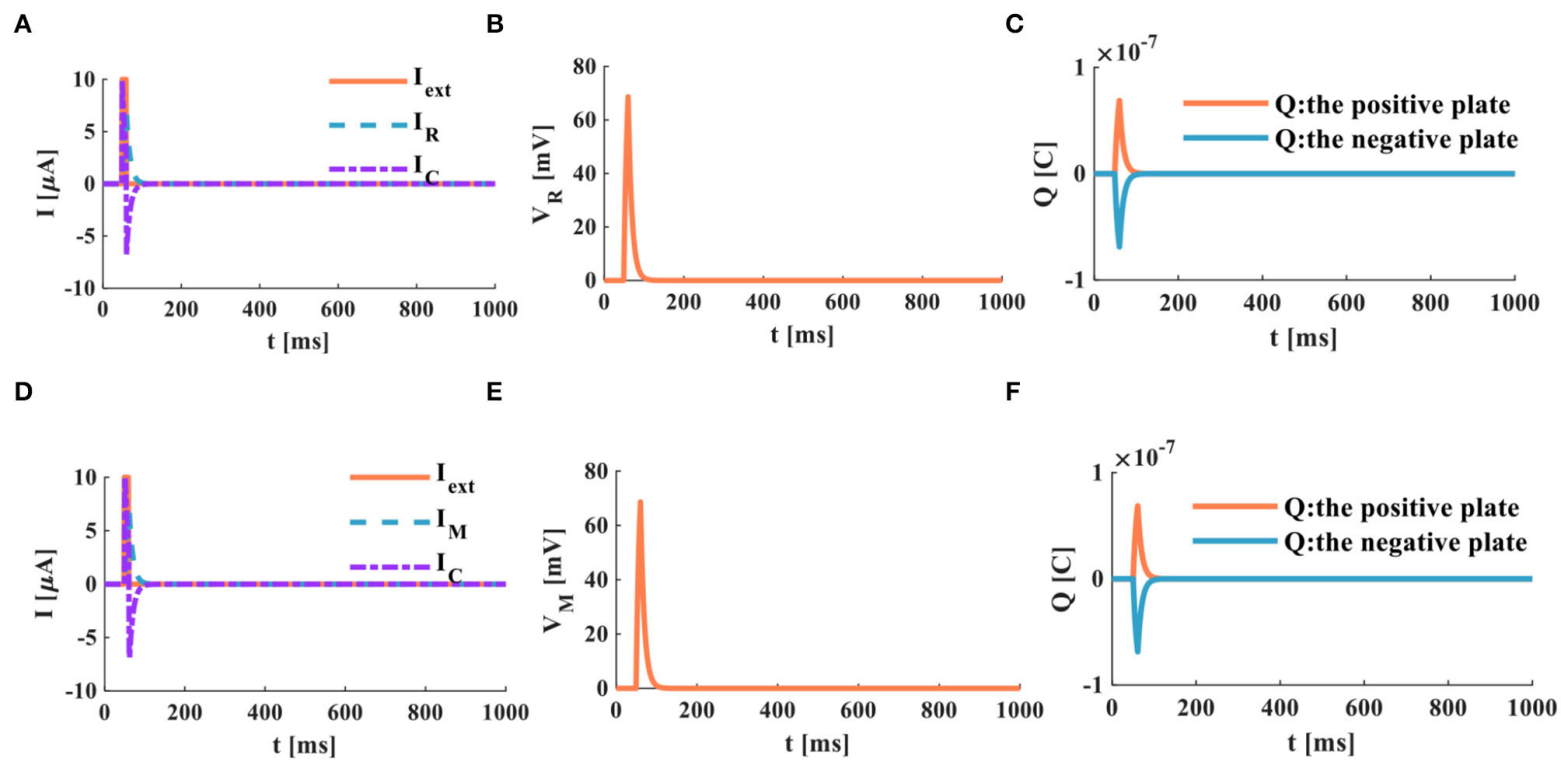
A single pulse acts on the RC and the MC neuron circuits, **(A)** the current flows through the resistor R and the capacitor C, **(B)** the membrane potential of the RC circuit, **(C)** the charges on the positive and negative plates in the RC circuit, **(D)** the current flows through the memristor M and the capacitor C, **(E)** the membrane potential of the MC circuit, **(F)** the charges on the positive and negative plates in the MC circuit.

The RC and MC neuron circuits generate the capacitor current and the resistor current (Figures 14A,D). The RC and MC neuron circuits produce the action potentials, which are closer to the firing behaviors of biological neurons (Figures 14B,E). When a single pulse appears, charges accumulate on the positive and negative plates of the membrane capacitor. When the single pulse disappears, charges of the positive and negative plates become zero (Figures 14C,F).

The simulation results indicate that the RC and MC neuron circuits are capable of generating action potential under appropriate conditions. The firing behaviors of biological neurons can be reproduced by the RC and MC neuron circuits. The action time of the external stimulus, the resistor (memristor), and capacitor values are the main factors to affect the production of the action potential.

## 4. The Synchronous and Asynchronous Phenomena in the RC and the MC Neuron Circuits

Most brain functions (memory, cognition, and perception) are realized by sharing information in collective neurons. Synchronous and asynchronous phenomena are nonlinear dynamic behaviors in the brain. The synchronous behaviors in the brain play a vital role in the connection between various brain activities and the firing patterns of neurons (Zerouali et al., 2013). To understand brain mechanisms and dynamics, we need to explore the synchronous and asynchronous activities in the brain.

**Figure 15:**
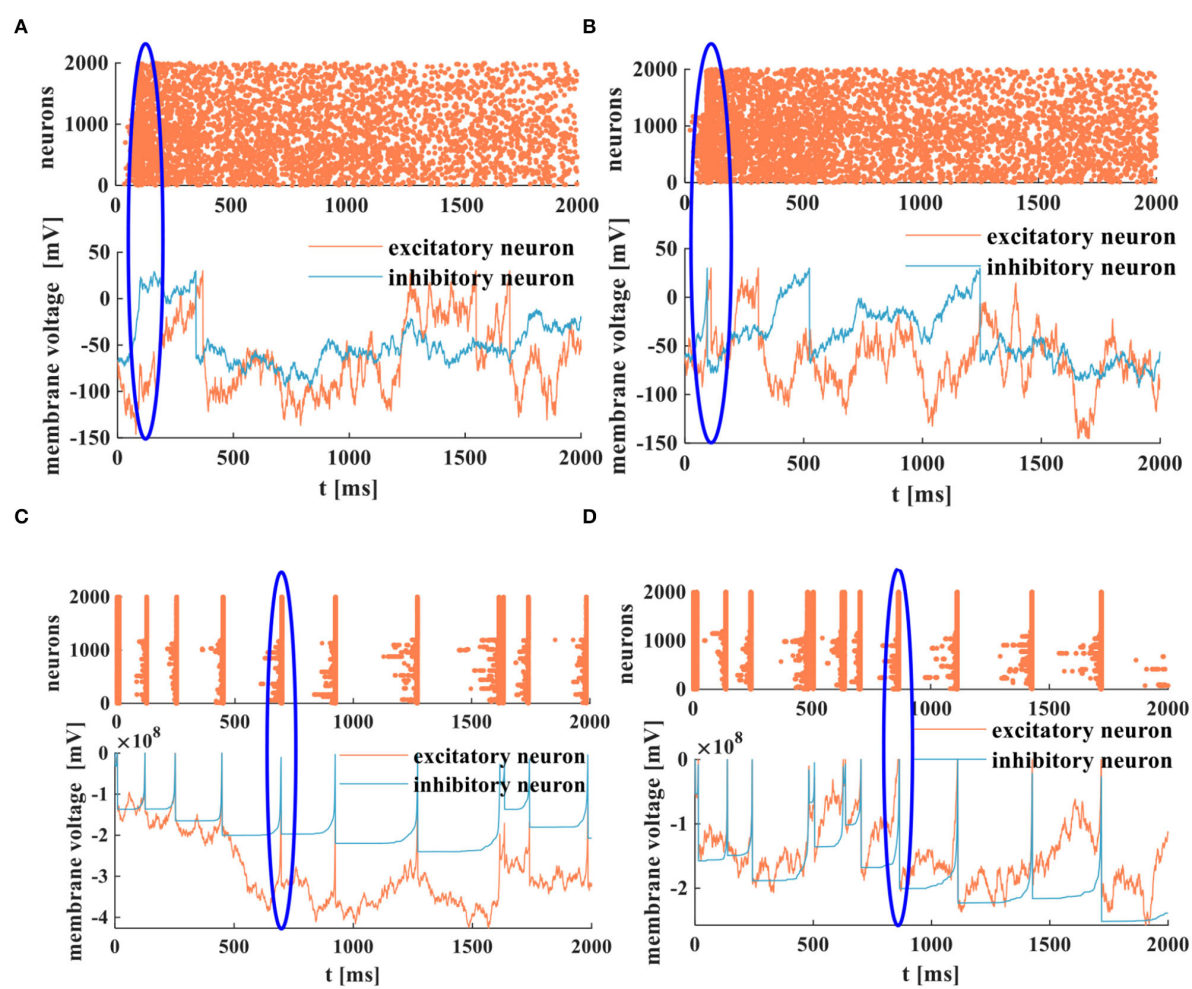
The spike raster and the membrane potentials of the RC and the MC neuron models, **(A)** the asynchronous behaviors and the membrane potential of the RC neuron model, **(B)** the asynchronous behaviors and the membrane potential of the MC neuron model, **(C)** the synchronous behaviors and the membrane potential of the RC neuron model, **(D)** the synchronous behaviors and the membrane potential of the MC neuron model.

We use the RC and the MC circuits to emulate the 2-D network of 2 × 10^3^ randomly coupled spiking neurons. The ratio between the excitatory and inhibitory neurons is set to be 4–1, which means 1,600 excitatory neurons and 400 inhibitory neurons in the neuron network. The simulation time is 2, 000*ms*, the resistance *R* = 10^3^Ω, and the capacitance *C* = 10^−6^*F*. The neuron activities in the neocortex are very irregular, and the action potentials are produced stochastically. Hence, we use random numbers as the external stimulus current to generate action potentials and the raster plots, as shown in Figure 15 (the corresponding parameter values and the program refer to Izhikevich, 2003):

The membrane potential peak value is 30*mV*. The action potentials are generated stochastically in the 2-D networks. The RC and MC neuron networks exhibit collective behaviors of neurons, and the asynchronous state of neuron activities is similar to the cortical-like asynchronous dynamics (Izhikevich, 2003). The blue oblate elliptical area performs the occasional synchronous behaviors in asynchronous activities (Figures 15A,B).

When the strength of the inhibitory input is reduced by two orders of magnitude, the RC and MC neuron circuits perform the synchronous behaviors [the thick vertical lines (coral color) corresponding to the action potential (blue and coral color)]. The blue oblate elliptical area is the corresponding relationship between synchronous behaviors and action potentials in neurons (Figures 15C,D). The generation of action potentials results from the synchronous activities of active neurons. As mentioned in Izhikevich (2003), the collective behaviors of neurons depend on the synapse strength and the external drive strength. The synchronous and asynchronous activities belong to the collective spiking dynamics of neurons, which is a way to explore the information propagation, processing, and interaction in the brain (Inagaki et al., 2021).

## 5. The Comparison of Memristive Neuron Circuits

After an overview of various neuron circuits, we select several novel research results to compare with the proposed MC neuron circuit for unique features, as shown in Table 1 (Amp represents the amplifier, T denotes the transistor, and GMMS means the generalized mean metastable switch).

**Table 1:** The performance comparison between memristive neuron circuits.

**Circuit name**	**MRLC**	**M-COMS**
Memrisotr type	Charge-controlled memristor	Ideal/nonideal memristor
Structure	R+L+C+M	1T1M+COMS
Capacitance Value	0.1 mF	-
Input/output	I(mA)/V(mV)	V(V)/I(μA)
Memristance range	-	2 × 10^4^Ω~2 × 10^6^Ω
Exhibited function	Spike and burst dynamics	Voltage loss compensation
	**MRA**	**MRC**
	AgInSbTe memristor	GMMS memristor
	R+M+3Amp	R+C+M
	-	5μF
	I(A)/V(V)	I(μA)/V(mV)
	–400Ω~400Ω	11Ω~4.6 × 10^5^
	Parallel programming operation	spike mode generation
	**MRCA**	**MC**
	Ag/AgInSbTe/Ta(ASIT) memristor	flux-controlled memristor
	R+C+M+2Amp	M+C
	0.2μF	1μF
	V(V)/V(V)	I(pA)/V(mV)
	0Ω~2 × 10^4^Ω	2 × 10^4^Ω~2 × 10^6^Ω
	Adaptive adjustment of firing rate	Action potential generation

We choose five neuron circuits for the comparison. The MRLC neuron circuit (Innocenti et al., 2021) consists of the resistor (R), the capacitor (C), the inductor (L), and the memristor (M). The charge-controlled HP memristor is chosen to realize the neuron circuit. It needs a large stimulus current to generate spiking and bursting phenomena in a neuron. The crossbar array of the 1T1M structure (Nguyen et al., 2021) is combined with CMOS to implement the M-COMS hybrid neuron circuit. Its wide memristance range is the same as that in the memristor-based neuron circuit. The MRA neuron circuit (Hong et al., 2019) needs a large voltage or current to activate the output response to realize the parallel programming operation, and it is the same as the M-COMS neuron circuit. The MRC neuron circuit (Ostrovskii et al., 2022) with the small capacitance needs the small stimulus current to obtain the spike mode. The memristance range of the MRCA neuron circuit (Shi and Zeng, 2018) is similar to that of the MC neuron circuit, but it needs a large stimulus voltage to generate the pulse voltage to adaptively regulate the firing rate.

Generally speaking, the MC neuron circuit has few components and parameters, and the memristor belongs to the nanometer device. These properties lead to the small scale of the circuit, which is conducive to the construction of large-scale neuron networks and large-scale circuit integration. The MC circuit is superior to other circuits: 1) the flux-controlled memristor coincides with the voltage-controlled ion channel in biological neurons. 2) The input-output form of I(pA)/V(mV) corresponds to the current input and the voltage output in biological neurons. 3) The capacitance value is close to that of cell membrane capacitance measured by biophysiological experiments. 4) The biological signal propagated between neurons is very weak, and the input current at the pA level is similar to the biological signal. These advantages help us to mimic the structure and function of neurons effectively and even achieve many biological neuron characteristics. Different types of neuron circuits realize the various functions. The MC neuron circuit focuses on the production of action potentials.

## 6. Conclusions

The RC and MC neuron circuits provide simple physical models to simulate biological neurons. They reproduce the firing behaviors of neurons and replicate the collective activities of neurons. We compared the traditional RC neuron circuit with the proposed MC neuron circuit, and both circuits could mimic the firing behaviors of neurons. The key advantage was found that the charge and discharge time of the MC neuron circuit was short. When the parameter values were appropriately chosen, the action time was sufficient, and the external stimulus was significant, the RC and MC neuron circuits could produce the action potential. Meanwhile, the RC and the MC circuits could emulate the synchronous and asynchronous behaviors of the collective neurons. The proposed MC neuron model has many characteristics, including simple structure, nanoscale, non-volatility, and easy-to-implement hardware circuit. It provides the possibility for the application of a memristor in biological neurology and electrophysiology.

## Data Availability Statement

The original contributions presented in the study are included in the article/supplementary material, further inquiries can be directed to the corresponding author.

## Author Contributions

XF built models and simulations, carried out the experimental analysis, and prepared the manuscript in this work. YT and FZ corrected the English writing and grammatical errors in the manuscript. SD and LW supervised the content of the manuscript and results of the simluations. All authors contributed to the article and approved the submitted version.

## Funding

Project supported by the National Key R and D Program of China (Grant No. 2018YFB1306600), the National Natural Science Foundation of China (Grant Nos. 62076207, 62076208, U20A20227), the Fundamental Science and Advanced Technology Research Foundation of Chongqing, China (Grant No. cstc2017jcyjBX0050).

## Conflict of Interest

The authors declare that the research was conducted in the absence of any commercial or financial relationships that could be construed as a potential conflict of interest.

## Publisher's Note

All claims expressed in this article are solely those of the authors and do not necessarily represent those of their affiliated organizations, or those of the publisher, the editors and the reviewers. Any product that may be evaluated in this article, or claim that may be made by its manufacturer, is not guaranteed or endorsed by the publisher.
